# Reactive microglia after taste nerve injury: comparison to nerve injury models of chronic pain

**DOI:** 10.12688/f1000research.2-65.v1

**Published:** 2013-02-28

**Authors:** Dianna L Bartel, Thomas E Finger

**Affiliations:** 1 Rocky Mountain Taste & Smell Center, Neuroscience Program, Department of Cellular and Developmental Biology, University of Colorado School of Medicine, Aurora, CO, USA

## Abstract

The chorda tympani (CT), which innervates taste buds on the anterior portion of the tongue, is susceptible to damage during inner ear surgeries. Injury to the CT causes a disappearance of taste buds, which is concurrent with significant microglial responses at central nerve terminals in the nucleus of the solitary tract (nTS). The resulting taste disturbances that can occur may persist for months or years, long after the nerve and taste buds have regenerated. These persistent changes in taste sensation suggest alterations in central functioning and may be related to the microglial responses. This is reminiscent of nerve injuries that result in chronic pain, where microglial reactivity is essential in maintaining the altered sensation (i.e., pain). In these models, methods that diminish microglial responses also diminish the corresponding pain behavior. Although the CT nerve does not contain nociceptive pain fibers, the microglial reactivity after CT damage is similar to that described in pain models. Therefore, methods that decrease microglial responses in pain models were used here to test if they could also affect microglial reactivity after CT injury. Treatment with minocycline, an antibiotic that dampens pain responsive microglia, was largely ineffective in diminishing microglial responses after CT injury. In addition, signaling through the toll-like 4 receptor (TLR4) does not seem to be required after CT injury as blocking or deleting TLR4 had no effect on microglial reactivity. These results suggest that microglial responses following CT injury rely on different signaling mechanisms than those described in nerve injuries resulting in chronic pain.

## Introduction

The chorda tympani (CT) nerve is the sensory branch of the seventh cranial nerve that innervates taste buds on the anterior tongue. The CT runs through the middle ear where is particularly susceptible to injury during ear surgeries ^1^. CT transection (CTx) is accompanied by a disappearance of taste buds on the denervated side of the tongue ^2, 3^. At the same time, significant microglial responses also occur in the first central gustatory relay – the nucleus of the solitary tract (nTS) ^4^. This nerve damage can cause a loss or distortion of taste, or dysguesia, that can persist for months or years long after the CT nerve and taste buds have regenerated ^5^. Such long-lasting dysguesias suggest alterations in central nervous function.

The central glial reactivity that occurs after damage to other sensory nerves actively contributes to abnormal sensations that arise after nerve damage. For example, increasing evidence from animal models suggests that microglial reactivity in particular is essential to initiate and maintain chronic pain (reviewed in ^6– 9^. This in large part explains why traditional pain drugs that directly target neuronal cells do not completely quiet persistent pain messages – because the neurons’ heightened sensitivity is also driven by microglia. A similar phenomenon might explain the lasting dysguesias after injury to the CT. Hence, understanding the mechanisms involved in microglial responses has important implications for treating abnormal sensations caused by nerve injury.

Even though the CT nerve does not contain any nociceptive C-fibers ^10^, which are themselves a source of microglial responses ^11, 12^, the general profile of the microglial responses are similar with CT injury as that seen in pain models. Specifically, damage to the CT causes significant microglial responses in terms of morphological reactivity and an increased density of microglial cells. Within a day after CT injury, microglia in the vicinity of afferent CT terminals changed their morphology from the characteristic ramified morphology to a hypertrophied reactive morphology, characterized by shorter thicker processes and an amoeboid shape. The increased microglial population primarily resulted from microglial proliferation, which was supplemented by microglial migration within sub-divisions of the nTS ^4^.

While the details of how nerve injury activates microglia are not entirely known, in nerve injuries that result in neuropathic pain pharmaceutical treatments can diminish pain behavior as well as the corresponding microglial responses. Therefore, various treatments and models that decrease microglial responses in experimental pain models were used here to test if they could dampen the microglial responses in the nTS after CT injury.

### Purinergic signaling (P2X _2_ and P2X _3_)

Microglia express multiple purinergic receptors. Upon sensing changes in the environment, this purinergic signaling results in several functional phenotypes such as process extension, migration, proliferation and phagocytosis (reviewed in ^13^). Here, we used mice that lack the P2X _2_ and P2X _3_ receptors to test whether these subunits are required for microglial responses following CT injury. Further, because the gustatory nerves of the P2X-dlbKO mice are not responsive to taste stimulation ^14^, this model also tested whether the disruption of normal gustatory signaling might alter microglial reactivity.

### Minocycline

This antibiotic is commonly used to treat chronic pain in animal models. Minocycline is a tetracycline derivative with anti-inflammatory and neuroprotective effects that are unrelated to its anti-microbial action (see reviews ^15, 16^). This compound is highly hydrophobic and readily crosses the blood-brain barrier. Multiple effects can account for the alleviation of pain behavior with minocycline, such as inhibition of matrix metalloproteinase as well as other anti-inflammatory, antioxidant and anti-apoptotic properties ^17– 19^. Minocycline treatment also results in reduced expression of the microglial marker Iba1 ^20^. Although this treatment has many modes of action, minocycline can only prevent but not reverse neuropathic pain ^21^. Therefore, to test if minocycline could diminish microglial reactivity in the nTS, the minocycline was administered to the animals before they received CTx and the animals continued to receive treatment for the duration of their survival.

### Toll-like receptor 4 (TLR4)

Recent studies have shown that TLR4 signaling is involved in nerve injury-induced microglial reactivity and related pain behavior ^22, 23^. Deletion of TLR4 can prevent pain and microglial reactivity from developing after spinal nerve injury ^22^. Hence, we tested whether TLR4 signaling is necessary for microglial reactivity after CT damage by performing CTx on the C3H mice that lack functional TLR4.

TLR4 signaling can also be inhibited with naloxone. Recent studies demonstrated that both the opioid antagonist (-)-naloxone and the non-opioid (+)-naloxone inhibit toll-like receptor 4 (TLR4) signaling and reverse neuropathic pain following spinal nerve injuries ^23, 24^. Hence, we performed CTx on the C57BL6/J animals that were then treated with naloxone as has been done in neuropathic pain models ^24^.

The current study used these methods and models and examined their effects on microglial reactivity in the nTS following CT injury. Specifically, we examined microglial morphology, counted the number of microglia and measured the fluorescent intensity of microglial markers.

## Materials and methods

### Animals

The experiments were conducted on male and female mice aged three to nine months (strains listed below and in Table 1). All animals were housed on a 14-hour light cycle with access to standard chow *ad-libitum*. The following protocols were approved by the Institutional Animal Care and Use Committees at the University of Colorado Anschutz Medical Campus (Aurora, CO).

**Table 1.** Number and genotype of animals used in experiments.

Experiments	Genotype	Number of animals
Purinergic signaling	P2X-dblKO P2X-WT C57BL6/(controls)	4 4 2
Minocycline	C57BL6/J	3 – Minocycline treated 6 – controls
TLR4 mutants Naloxone	C3H C3HeB C57BL6/J (controls) C57BL/J	3 3 3 15 – Naloxone treated 3 – controls

## Mouse strains

C57BL6/J – These mice were originally obtained from The Jackson Laboratory (Bar Harbor, ME) and were bred in-house. A total of 32 C57BL6/J animals were used in these experiments.

P2X _2_/P2X _3_^-/-^ – These double-knockout mice (P2X-dblKO) and their wild-type controls (P2X-WT) were originally obtained from Roche Palo Alto (Palo Alto, CA; ^25^) and were subsequently bred at Charles River Transgenics Laboratory or in-house at the University of Colorado. The gustatory nerves in these mice are unresponsive to taste stimuli ^14^. A total of four P2X-dblKO animals and four P2X-WT animals were used in these experiments.

C3H/HeJ ( *Tlr4 ^Lps-d^*) – The C3H mice carry a point mutation in the cystolic domain of the Toll-like receptor 4 protein (TLR4), resulting in the exchange of amino acid 712 from proline to histidine that disrupts the functionality of the receptor ^26^. These mice and their wild-type controls (C3HeB/FeJ) were obtained from The Jackson Laboratory. A total of three C3H animals and three C3HeB animals were used in these experiments.

### Surgical procedures

The chorda tympani transection and perfusion procedures were performed according to methods detailed in a previous publication ^4^ and are described briefly here.

***CT transection (CTx)*:** The animals of the different treatment groups (a total of 41 mice) were anesthetized with intramuscular injections of dexmedetomadine hydrochloride (0.4mg/kg; Pfizer, Finland) and ketamine hydrochloride (40mg/kg; Bioniche Pharma; Lake Forest, IL). After placing the animal in a non-traumatic head holder, we visualized and transected the CT using the transaural approach on the right side. The left CT remained intact to serve as an internal control. The anesthesia was then reversed with antipamezole (2mg/kg: Pfizer, Finland), the antidote to dexmedetomadine. The animals survived for 3 days.

***Perfusion and tissue fixation*:** The animals were deeply anesthetized with 50mg/kg sodium pentobarbital (Ovation Pharmaceuticals Inc; Deerfield, IL) and perfused transcardially with 15 mL of 0.9% sodium chloride followed by 25 mL of 4% paraformaldehyde (PFA in 0.1M phosphate buffer). The brainstem was dissected from the skull, which was then postfixed in PFA for 4 hours at room temperature and cryoprotected with 20% sucrose in 0.1M phosphate buffer overnight at 4°C. The next day, the tissue was mounted in OCT compound (Fisher Scientific; Pittsburgh, PA) and cut on a cryostat. Free-floating 40 µm sections were collected into PBS (0.1M phosphate buffered saline).

### Immunohistochemistry

After three ten-minute washes in PBS, tissue sections were incubated for one hour in blocking solution [1% bovine serum albumin (Sigma, St. Louis, MO), 0.3% triton (American Bioanalytical, Natick, MA) and 2% normal goat serum (Jackson Immunoresearch, West Grove, PA) in PBS]. Incubation with rabbit anti-Iba1 (Wako; Richmond, VA; polyclonal; #19-19741, dilution 1:500) or rat anti-cd11b (Serotec; Raleigh, NC; monoclonal; #MCA74GA, dilution 1:50) antibody diluted in blocking solution was carried out overnight at 4°C. The next day, three PBS washes were followed by 2 hours of incubation with the appropriate secondary antibody, Alexa488 goat anti-rabbit or Alexa488 goat anti-rat (Invitrogen, Carlsbad, CA; dilution 1:400 in blocking solution). For the final 25 minutes, green fluorescent Nissl stain (NeuroTrace; Molecular Probes, Eugene, OR) was added to the incubation solution at 1:100. After the sections were washed several times in buffer, they were mounted onto slides and coverslips were applied with Fluormount G (Southern Biotechnology Associate, Inc.; Birmingham, AL).

### nTS identification

**Figure 1.**
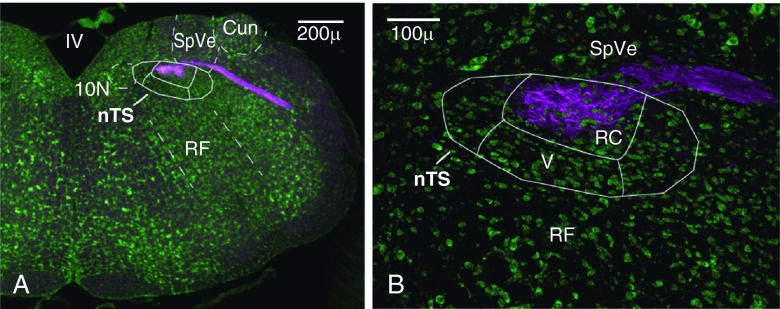
Staining for the purinergic P2X ^2^ receptor delineates afferent chorda tympani nerve (CT) projections. **A**. Borders of the nTS and surrounding nuclear regions were identified with fluorescent Nissl staining (green). The P2X ^2^ staining in magenta delineates the area of incoming CT fibers. **B**. The majority of CT projections terminate within the rostral central subnucleus (RC) though some finer fibers extend into the ventral subnucleus (V). Images in all figures are displayed in the orientation shown above (medial - left, dorsal - top). Cun - external cuneate, nTS - nucleus of solitary tract, 10N - dorsal motor nucleus of vagus, SpVe - spinal vestibular nucleus, RF - reticular formation, IV - fourth ventricle.

The densest projections of chorda tympani fibers occur in rostral levels of the nTS, designated R1, R2 and R3 ^4, 27^. Using the fluorescent Nissl stain, we could delineate the rostral central (RC) and ventral (V) subdivisions of the nTS according to previous studies ( ^28, 29^ and see Figure 1). The borders of the nTS were outlined on grayscale fluorescent Nissl images in Adobe Illustrator 10 and superimposed onto the color images. All representative images shown in figures are taken at levels R2 or R3.

### Image acquisition and analysis

Confocal images were acquired with an Olympus Fluoview Laser Scanning Confocal Microscope using 20× oil-immersion objective (N.A. 0.80) or 60× oil-immersion objective (N.A. 1.4). The green and red channels were obtained sequentially and merged together to prevent sideband excitation of the fluorophores.

Images that were used for counting microglial cells were obtained with the same acquisition parameters (i.e., laser intensity, gain and offset) on the lesioned and unlesioned sides to permit quantitative comparisons. Cell counts were done as previously described ^4^. Briefly, using the optical dissector method a pair of parallel images spaced 5 µm apart was used to count Iba1+ microglia. A microglial cell body was counted in the ‘reference’ image if the same cell body was not present in the partner ‘look-up’ image.

Conventional epifluorescent images were obtained using an Olympus BX41 upright microscope and a 20× objective (N.A. 0.50). These images were used to measure fluorescence levels of Iba1 and cd11b. Using the ImageJ version 1.62 software (National Institutes of Health; Bethesda, MD), fluorescence levels were measured within a defined circular region of interest (ROI=1,596 40 µm ^2^). For each animal, four ROI measurements were taken on intact and transected sides in the following regions: rostral central nTS (RC), ventral nTS (V) and spinal vestibular nucleus (SpVe).

The cell counts and intensity measurements for each experiment were analyzed using SPSS software (Chicago, IL) with appropriate ANOVAs and Tukey’s post-hoc analysis. Significance was defined as p<0.05.

### P2X-dblKO experiments

To test if P2X _2_ and P2X _3_ receptors are necessary for microglial responses, CTx was performed on four P2X-dblKO mice and four P2X-WT mice. Microglia were stained with Iba1, imaged and then counted.

### Minocycline experiments

To test if minocycline could decrease microglial responses after CTx, three C57BL6/J mice received minocycline before and after CTx. Minocycline hydrochloride (Sigma M9511) was administered via intraperitoneal injection at 50mg/kg every 12 hours based on previous studies ^17, 21, 30^. Treatment began one day prior to CTx surgery and continued until the animal was sacrificed three days later. Microglia were stained with Iba1 and cd11b and imaged to quantify cell numbers and levels of fluorescence.

### TLR4 experiments

***TLR4 mutants (C3H mice)*:** These experiments tested whether a functional TLR4 was required for the nerve damaged-induced microglial response. CTx was performed on three C3H mice, which lack a functional TLR4, as well as three of their wild-type siblings (C3HeB) to compare to the C57BL6/J animals. The animals survived for three days. Microglia were stained with Iba1, imaged and then counted.

***Naloxone*:** These experiments sought to block TLR4 with naloxone treatment at the onset of the CT injury. A total of eight C57BL/6 animals received CTx and were then treated with naloxone hydrochloride (Sigma, #N7758, racemic mixture; administered subcutaneously), seven C57BL/6 animals were treated with naloxone but did not received CTx. Naloxone has a short half-life of ~60 minutes in the serum ^31^. To examine whether naloxone could prevent the microglial response *days* after CTx, it was necessary to administer the naloxone continuously, which was achieved by means of osmotic minipumps. The total doses of naloxone, 10mg, in these experiments is comparable to previous studies on rats using chronic intrathecal and acute subcutaneous administration ^23^ and other studies on mice using chronic subcutaneous administration ^32^.

The ALZET® pump model 1003D (delivers 1 μl/hr, up to 3 days) was used in these experiments. The pumps were filled according to company instructions. So as not to delay the delivery of drug, prior to implantation, the pump was allowed to reach its steady-state pumping rate by incubating in sterile saline at 37°C for 6–12 hours.

According to the manufacturer, the solubility of naloxone is 50mg/mL. Since the total volume of the pumps is 100 μl, the total amount of naloxone that could be administered by one pump was 5mg. This dosage of naloxone was as follows:

5mg/0.02kg/3dy = 1.67mg/0.02kg/1dy = 69.58μg/0.02kg/1hour

69.58μg/0.02kg = 0.06958mg/0.02kg = 3.48mg/kg/1hour

To achieve the highest dosage of naloxone (10mg), the animals received two of these pumps for three days. Hence, the dose of naloxone administered was 6.96mg/kg/1hour.

After the animal was sedated, the animal was given a starter injection of the naloxone solution at the same concentration/day determined to be delivered by the pump (i.e., in the example above 1.67mg/0.02kg). This ‘booster’ shot was given via intraperitoneal (I.P.) injection in order to get naloxone to an effective level before nerve injury. To insert the pump, the area on the back of the neck between the scapulae was shaved and surgically prepped. After making a ~1-cm incision in the skin, a hemostat was inserted under the skin, then gently opened and closed to create a pocket for the pump. The pump was inserted into the pocket, exit port first, and the incision was closed with several stitches with Decon suture. While the animal was still sedated, the CT was transected.

## Behavior testing with naloxone treatment

We first wanted to test whether naloxone was gaining access to the CNS. Previous studies have shown that naloxone causes a decreased intake of preferred foods such as sweet-tasting chow or solutions ^33– 37^. Therefore, we tested the animals’ sweet preference as an indication of the effectiveness of the naloxone treatment. Sweet preference was measured using a two-bottle taste preference test in which the animals are given a choice between water and sucrose. The preference score was calculated [sucrose (mL)/total fluid (mL)] such that the higher the score the greater the sweet preference with 0.5 representing no preference. The general protocol involved training the animals for several days with the two bottles before they received naloxone pumps. At the end of each training and testing period, fluid intake was recorded. Several different testing parameters were used in these experiments and are described as follows.

## Behavior testing with naloxone *and* CTx

The following describe experiments that used naloxone treatment (10mg) *and* CTx.

A. Mice were given continual access to two-bottles: one with water and one with 300mM (10%) sucrose where the bottles were switched sides every 24 hours at which time the fluid intake was recorded. Mice were trained for 4 days before receiving pumps with 10mg Naloxone or saline and CTx. The mice recovered for 6 hours with full access to water and were then again given the two bottles with water and sucrose for 2 days and were then sacrificed.

B. Mice were placed on a 22.5 hour water deprivation schedule and were trained for 3 days during which they had access to fluids for a total of 1.5 hours per day: during the first 30 minutes both bottles contained water, then mice were given one bottle of water and one bottle of 300mM (10%) sucrose for 60 minutes during which bottles were switched sides after 30 minutes. On day 4, pumps filled with 10mg naloxone or saline were implanted followed by CTx. The mice recovered for 6 hours with full access to water and were then tested with water and sucrose for 3 days and were then sacrificed.

## Behavior testing with naloxone only

To ensure that CTx was not affecting the taste preference behavior, animals that treated with 10mg of naloxone but did *not* undergo CTx were also tested. The concentration of sucrose was also lessened in these experiments to ensure that more subtle effects of NLX could be detected.

A. Mice were given continual access to two-bottles: one with water and one with 200mM (7%) sucrose where the bottles were switched sides every 24 hours at which time the fluid intake was recorded. Mice were trained for 2 days before receiving pumps with 10mg naloxone. The mice recovered for 6 hours with full access to water, were tested with water and sucrose for 2 days and then sacrificed.

B. Mice were water restricted for 21.5 hours and were trained for 2 days during which they had access to fluids for a total of 3.5 hours per day: during the first 90 minutes both bottles contained water, then mice were given one bottle of water and one bottle of 200mM (7%) sucrose for 2 hours during which bottles were switched sides after 60 minutes. On day 3, pumps filled with 10mg naloxone were implanted. The mice recovered for 6 hours with full access to water, were tested with water and sucrose for 2 days and then sacrificed.

## Results

**Figure 2.**
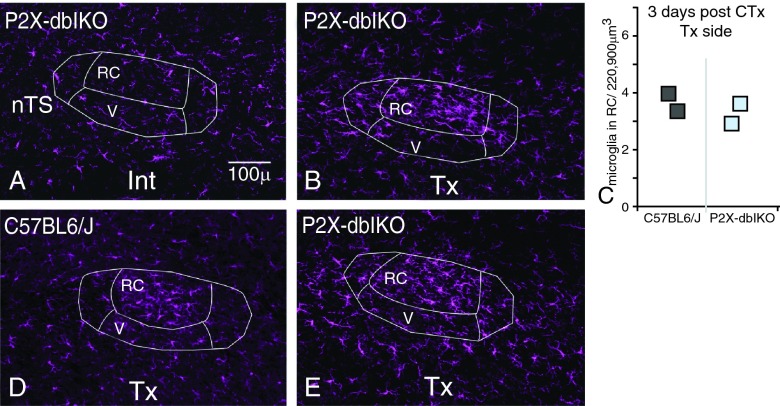
Reactive microglia appear in the nTS following CT transection (CTx) in the P2X-dblKO animals. **A**. As revealed with Iba1 staining, microglia on the intact side (Int) of the nTS occupy distinct spatial areas throughout the nTS and surrounding areas. **B**. At 3 days post CTx on the transected side (Tx), a dense cluster of Iba1 immunoreactivity appears in the RC subnucleus of the nTS and microglia display reactive morphologies. **C**. The number of Iba1 labeled microglia in the RC subnucleus on the Tx side in the P2X-dblKO animals (light blue squares) does not appear to differ from wild-type animals (dark grey squares) at 3 days post CTx. Each square represents the mean from one case. **D**. At 10 days post CTx, the cluster of reactive microglia is still present in the RC subnucleus of C57BL6/J mice. **E**. Similarly, reactive microglia remain at 10 days post CTx in the P2X-dblKO animals.

Staining for P2X _2_ and P2X _3_ receptors in the rostral medulla delineates the incoming CT terminations within the nTS ^4^. As shown in Figure 1, P2X _2_ staining in magenta identifies terminal CT fibers, which are primarily contained within the rostral central (RC) subnucleus of the nTS. The RC subnucleus is also where reactive microglia appear following CT injury ^4^ and (see Figure 2).

### Microglial responses in P2X-dblKO mice

To test whether P2X _2_ and P2X _3_ receptors are necessary for microglial reactivity in the nTS, we performed CTx in mice that lacked these functional receptors (P2X-dlbKO). In both the P2X-WT and the P2X-dblKO cases, Iba1+ microglia on the unlesioned intact side (Int) of the nTS were evenly distributed throughout the tissue ( Figure 2A shows the Int side of P2X-dblKO). By the third day after injury, a dense cluster of Iba1 immunoreactivity appeared in the nTS on the transected side (Tx). Specifically, this aggregate of Iba1+ microglia was primarily confined to the RC subnucleus, which contains the densest projections of CT terminal fibers ( Figure 2B shows Tx side of P2X-dlbKO). These microglial cells displayed reactive morphologies with thickened processes and enlarged somata. This result parallels the course of microglial responses following CTx in C57BL6/J animals ^4^. Further, the number of microglial cells in the RC subnucleus in P2X-dblKO did not differ from the number of microglia in their wild-type siblings ( Figure 2C). That is, the density of microglia increased in the RC subnucleus in the P2X-dblKO, just as seen in the wild-type siblings as well as C57BL6/J animals. At ten days post CTx, the cluster of reactive microglia remains in the RC subnucleus in C57BL6/J animals ( Figure 2D). Similarly, reactive microglia are also present at 10 days after CTx in the P2X-dblKO animals ( Figure 2E). Hence, even in the absence of P2X _2_ and P2X _3_ transection of the CT still resulted in reactive microglia.

### Minocycline experiments

**Figure 3.**
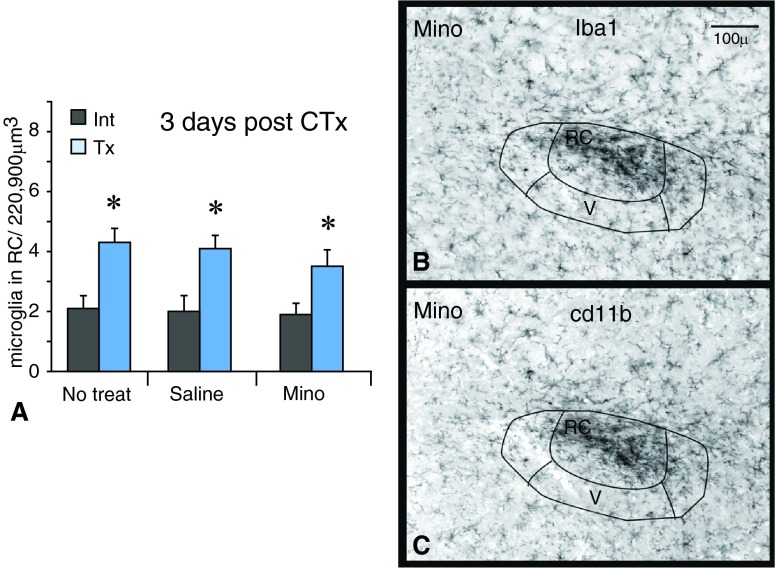
Minocycline does not affect microglial numbers after CTx. **A**. The number of Iba1-labeled microglia in the RC subnucleus increases on the transected sides (Tx) compared to the intact sides (Int) with ‘No treatment’ and saline treatment. The number of microglia also increases on the transected side with minocycline treatment (Mino). An asterisk (*) denotes a significant difference on the transected side compared to the corresponding intact side with p<0.05, SEM error bars, n=3 for each group. **B,C**. After minocycline treatment, staining for Iba1 and cd11b reveals the typical cluster of reactive microglia in the RC subnucleus on the transected sides.

In minocycline treated cases, microglial cells still reacted throughout the CT terminal field. At three days after injury, the number of microglia in the RC subnucleus still increased on the transected sides compared to the intact sides whether the animals received minocycline, saline or no treatment ( Figure 3A; n=3 each). Microglia were also morphologically reactive in the RC subnucleus, as seen when labeled with Iba1 or cd11b ( Figure 3B,C).

Number of microglia in the nTS three days post chorda tympani transection in mice treated with minocycline, saline or no treatment The number of microglia in the rostral central (RC) subnucleus of the nTS at three days post chorda tympani transection (CTx) in mice treated with minocycline (Mino), saline or no treatment. Three animals from each treatment were analyzed. From each animal, six microglial counts were taken throughout the rostral levels on both intact (Int) and transected (Tx) sides. The cell counts were analyzed using SPSS software with appropriate ANOVAs and Tukey’s post-hoc analysis. Significance was defined as p<0.05.

**Figure 4.**
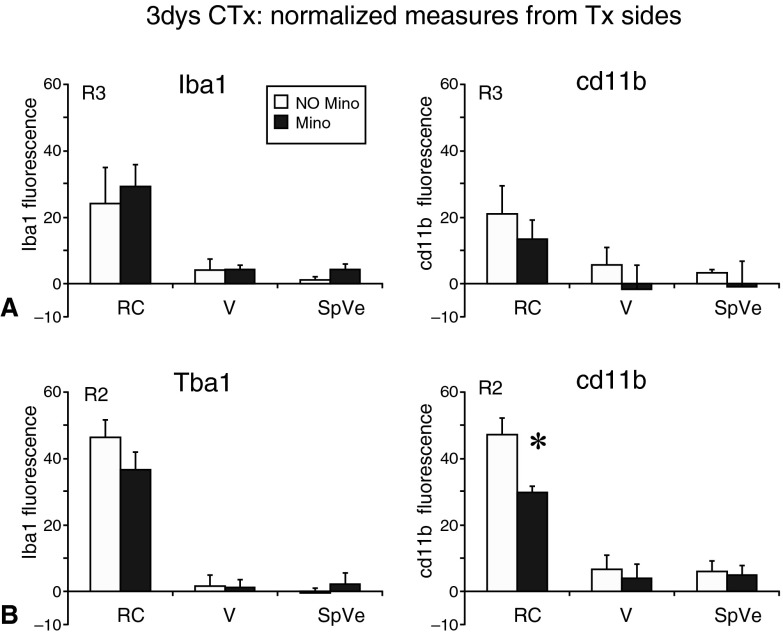
Minocycline results in lower levels of cd11b but does not affect Iba1. Fluorescent levels were measured on both intact and transected sides. In order to normalize the levels on the transected side (shown in the figure), the levels from the intact sides were subtracted from the transected sides. **A**. At the rostral level R3, minocycline treatment (dark bars) did not affect the intensity of Iba1 or cd11b staining compared to no treatment (white bars). The fluorescent intensity in the rostral central (RC) and ventral (V) subregions of the nTS as well as the neighboring spinal vestibular nucleus (SpVe) is not affected by minocycline treatment. **B**. At the rostral level R2, minocycline treatment did not affect Iba1 levels but resulted in significantly lower fluorescence levels of cd11b in the RC subnucleus, though the levels in V and SpVe were not affected. The asterisk (*) denotes a significant difference on the transected side compared to the intact side with p<0.05, SEM error bars, n=3 for each group.

Since the number of microglial cells was similar after injury with or without minocycline treatment, we compared the levels of expression of two microglial markers: Iba1, a cytoplasmic calcium binding protein, and cd11b, a membrane-bound integrin receptor involved in phagocytosis. At the R2 and R3 rostral levels of the nTS ^27^, we measured the intensity of Iba1 and cd11b staining on the intact and transected sides. The fluorescent levels on the transected sides were normalized by subtracting the fluorescent levels from the corresponding intact sides. These normalized measurments were compared between no treatment (white bars) and minocycline treatment (dark bars; Figure 4). At level R3, minocycline did not affect the levels of Iba1 staining [F(1,4)=1.1, p=0.3] or cd11b staining [F(1,4)=10, p>0.05] in the rostral central (RC) or ventral (V) subnuclei of the nTS nor the immediately adjacent spinal vestibular region (SpVe; Figure 4A). However, at level R2 minocycline treatment did result in significantly decreased levels of cd11b but only in the RC [F(2,8)=23.9, p<0.05] (see Figure 4B). The fact that only the levels of cd11b, an intergrin receptor involved in phagocytic activity, were decreased with minocycline treatment might suggest that reactive microglia in the nTS are involved in phagocytosis.

Iba1 and cd11b fluorescence levels three days post chorda tympani transection in mice treated with minocycline or not treated Fluorescence levels of Iba1 and cd11b at three days post chorda tympani transection (CTx) in animals that were treated with minocycline (Mino) and animals that were not treated (No Mino). Three animals for each treatment, Mino and No Mino, were analyzed. The fluorescent intensity of Iba1 and cd11b were measured within a defined circular region of interest (ROI=1,596 40 µm2) in ImageJ. For each animal, two ROI measurements were taken at levels R2 (sheet 1) and R3 (sheet 2) on the intact (Int) and transected (Tx) sides in the following regions: rostral central (RC) subnucleus of the nTS, ventral (V) subnucleus of the nTS and the spinal vestibular nucleus (SpVe). The fluorescent levels on the transected sides were normalized by subtracting the fluorescent levels from the intact sides. These normalized values, which were used to generate the graphs shown in Figure 4, were then analyzed using SPSS software with the appropriate 2-way ANOVAs and Tukey’s post-hoc analysis where significant differences were defined as p<0.05.

### TLR4 experiments

**Figure 5.**
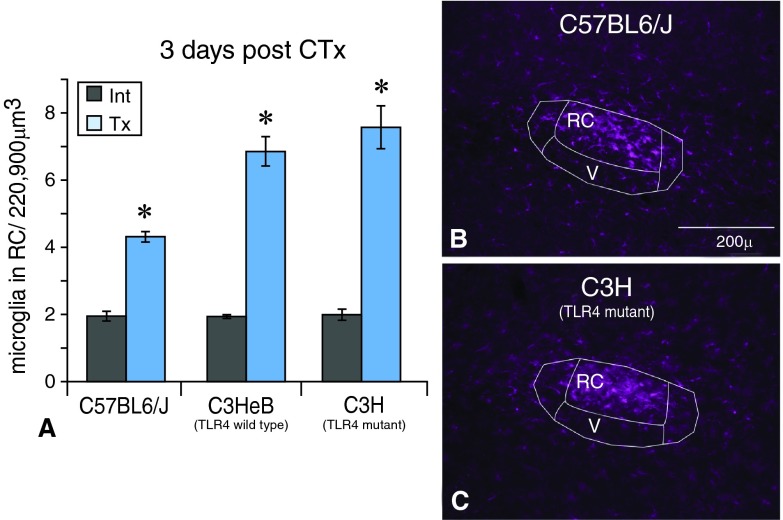
Microglia are morphologically reactive and increase in number after CTx in the TLR4 mutant animals. **A**. The number of Iba1+ microglia in the rostral central (RC) subnucleus of the nTS increases on the transected (Tx) side compared to the intact (Int) side in the C3H (TLR4 mutant) animals, as seen in C3HeB and C57BL6/J wild-type animals. An asterisk (*) denotes a significant difference on the transected side compared to the corresponding intact side with p<0.05, SEM error bars, n=3 for each group. **B,C**. Further, as seen in C57BL6/J wild-type animals the typical cluster of reactive Iba1+ microglia also appears in the RC subnucleus on the transected side in C3H (TLR4 mutant) animals.

***TLR4 mutants (C3H)*:** To test the possibility that TLR4 signaling is essential for microglial activation, CTx was performed on the TLR4 mutant animals (C3H) as well as their wild-type siblings (C3HeB). The typical cluster of Iba1-labeled microglial cells appeared in the RC subnucleus on the transected side in the TLR4 mutant animals just as seen in wild type animals ( Figure 5A,B). Microglia on the intact sides did not change (not shown). Further, the density of microglial cells also increased on the transected side compared to intact sides in the TLR4 mutant animals ( Figure 5C) just as in wild type controls. Thus, these data suggest that TLR4 signaling is not required for microglial activity after CT injury.

Number of microglia in the nTS three days post chorda tympani transection in C57BL6/J, C3HeB and C3H mice The number of microglia in the rostral central (RC) subnucleus of the nTS at three days post chorda tympani transection (CTx) in C57BL6/J (wild-type), C3HeB (TLR4 wild-type) and C3H (TLR4 mutant) animals. Three animals from each genotype were analyzed. From each animal, six microglial counts were taken throughout the rostral levels on both intact (Int) and transected (Tx) sides. The cell counts were analyzed using SPSS software with appropriate ANOVAs and Tukey’s post-hoc analysis. Significance was defined as p<0.05.

***Naloxone*:** These experiments were performed in C57BL6/J animals and sought to block TLR4 signaling with naloxone treatment at the onset of the CT injury.

## Behavior testing with naloxone and CTx

We first sought to test whether naloxone was affecting functionality in the CNS. In other studies on rats, the administration of naloxone causes a decreased intake of preferred foods such as sweet-tasting chow or sugar solutions ^33– 37^. Here, we determined the animals’ sweet preference score as an index of naloxone effectiveness.

**Figure 6.**
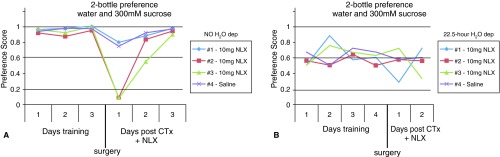
Naloxone (NLX) treatment does not decrease the intake of sucrose after CTx. **A**. When mice had continual access to water and sucrose, the animals preferred the sucrose solution to water during the three-day training period. The preference score was calculated [sucrose (mL)/total fluid (mL)] such that the higher the score indicates the greater the sweet preference with 0.5 representing no preference (each line represents an individual animal). After surgery to implant naloxone or saline pumps followed by CTx, the mice that received 10mg naloxone still preferred sucrose on the whole. **B**. When mice were water deprived for 22.5 hours with access to water and sucrose the remaining 1.5 hours, all mice preferred the sucrose solution before and after surgery (each line represents an individual animal).

During the training period, animals preferred the sucrose solution whether they were water deprived or not ( Figure 6). The preference score was calculated [sucrose (mL)/total fluid (mL)] so that the higher the score the greater the sweet preference. Hence, a score > 0.5 is indicative of sucrose preference. After placement of minipumps with 10mg naloxone or saline followed by CT injury, the overall sucrose preference in naloxone-treated animals did not differ from saline control animals. Although two naloxone treated animals showed a decreased intake of sucrose the first day after treatment ( Figure 6A, red and green lines), this behavior was not maintained. On the whole, the preference for sucrose did not consistently decrease in naloxone-treated animals that also underwent CTx.

Two-bottle preference experiments with water and 300mM sucrose before and after chorda tympani transection and treatment with naloxone Two-bottle preference experiments with water and 300mM sucrose before and after chorda tympani transection (CTx) and treatment with naloxone (NLX). In the first set of experiments in Figure 6A, four mice had continual access to the two bottles (water and sugar solution). The animals were trained for three days before undergoing CTx and receiving naloxone or saline treatment (post). For each day, the total amounts of each liquid consumed were used to calculate the preference score [sucrose (mL)/total fluid (mL)]. The higher the score indicates the greater the sweet preference with 0.5 representing no preference. In the second set of experiments in Figure 6B, another four mice were placed on a 22.5 hour water deprivation schedule where they had access to bottles of water and sugar solution the remaining 1.5 hours per day. The animals were trained for four days before undergoing CTx and receiving naloxone or saline treatment.

## Behavior testing with naloxone only

**Figure 7.**
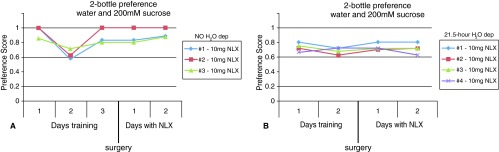
Naloxone (NLX) treatment alone does not decrease the intake of sucrose. **A**. When mice had continual access to water and sucrose, the animals showed less preference for sucrose on the second day of training (each line represents an individual animal). Because the animals had not yet received naloxone, this was likely a disturbance during the experiment. However, after naloxone treatment the animals still prefer sucrose solution to water. The preference score was calculated [sucrose (mL)/total fluid (mL)] such that the higher the score indicates the greater the sweet preference with 0.5 representing no preference (each line represents an individual animal). **B**. Similarly, when mice were water deprived for 21.5 hours with access to water and sucrose the remaining 2.5 hours, they maintained their preference for sucrose after treatment with 10mg naloxone (each line represents an individual animal).

To test if CTx might be interfering with the animals’ sweet preference behavior, the animals were tested with naloxone treatment only. When animals were not water deprived, there was a significant decrease in the intake of sucrose on the second day of training ( Figure 7A). As the mice had not yet received treatment, this suggests a disturbance or distraction during the training session. The next training day, mice resumed their intake of sucrose. However, after treatment with 10mg of naloxone the mice continued to prefer sucrose (i.e., scores near 1). Similarly, when animals were water deprived and only had access to the sucrose and water bottles for a few hours a day, treatment with 10mg naloxone did not cause a significant and consistent decrease in intake of sucrose solution ( Figure 7B).

Two-bottle preference experiments with water and 200mM sucrose before and after treatment with naloxone Two-bottle preference experiments with water and 200mM sucrose before and after treatment with naloxone. In the first set of experiments in Figure 7A, three mice had continual access to water and sugar solution. The animals were trained for three days before receiving naloxone treatment. For each day, the total amounts of each liquid consumed were used to calculate the preference score [sucrose (mL)/total fluid (mL)] such that the higher the score indicates the greater the sweet preference with 0.5 representing no preference. In the second set of experiments in Figure 7B, four mice were placed on a 22.5 hour water deprivation schedule where they had access to bottles of water and sugar solution the remaining 1.5 hours per day.

In summary, 10mg of naloxone administered subcutaneously by minipumps did not cause a reduced intake in sucrose. This behavior had not been affected by CT injury, since the naloxone-treated animals with and without CTx showed similar taste preference behavior. This suggests that naloxone was not accessing the brain at a sufficient level to affect this neuronally mediated taste behavior.

## Microglial studies with naloxone treatment

**Figure 8.**
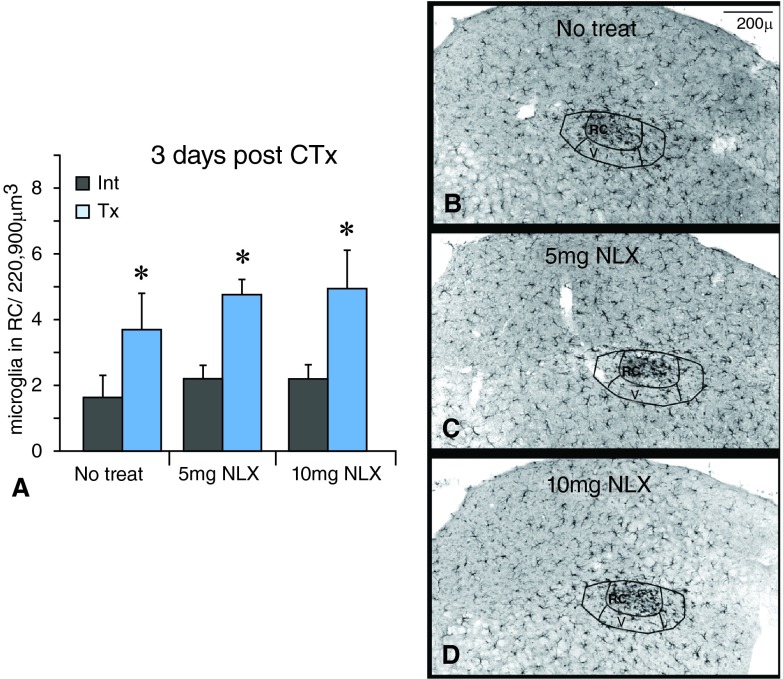
Naloxone (NLX) treatment does not affect microglia after CTx. **A**. The number of microglia in the rostral central (RC) subnucleus increases on the transected side (Tx) compared to the intact side (Int) when treated with naloxone. An asterisk (*) denotes a significant difference on the transected side compared to the corresponding intact side with p<0.05, SEM error bars, n=3 for each group. **B**. At 3 days after CTx and ‘No treatment’ Iba1 staining reveals reactive microglia in the RC subnucleus on the transected side. **C,D**. Similarly, reactive microglia are present in the RC subnucleus in mice treated with 5mg or 10mg NLX.

In naloxone-treated animals that received CTx, microglia still reacted throughout the CT terminal field at 3 days post injury. The typical cluster of Iba1 labeled microglial cells appeared in the RC subnucleus ( Figure 8B–D). Further, the number of microglial cells on the transected side after nerve injury remains increased in the RC subnucleus with naloxone (naloxone; blue bars) treatment compared to no treatment (grey bars; Figure 8A). Thus, naloxone treatment did not affect microglial reactivity in the nTS in terms of morphology or increased density. This suggests that naloxone-sensitive TLR4 signaling is not required for microglial activity in the nTS.

Number of microglia in the nTS three days post chorda tympani transection in mice treated with naloxone The number of microglia in the rostral (RC) subnucleus of the nTS at three days post CTx in mice treated with naloxone (NLX). Three animals from each treatment group (0mg, 5mg, 10mg) were analyzed. From each animal, six microglial counts were taken throughout the rostral levels on both intact (Int) and transected (Tx) sides. The cell counts were analyzed using SPSS software with appropriate ANOVAs and Tukey’s post-hoc analysis. Significance was defined as p<0.05.

## Discussion

The current study sought to elucidate the mechanisms involved in microglial responses following chorda tympani transection (CTx). These experiments were designed to address different possibilities of microglial activation in the nTS.

### P2X-dblKO

Using the P2X-dlbKO mice, we tested whether the P2X _2_ and P2X _3_ receptors were necessary for microglial reactivity with CTx. Microglial cells in the nTS increased in number and were morphologically reactive in the P2X-dblKO animals just as seen in wild type animals. These experiments suggest that these purinergic receptors are not required for microglial reactivity in the nTS.

Interestingly, the gustatory nerves of the P2X-dlbKO mice are not responsive to taste stimulation ^14^. Hence, the fact that microglial reactivity still occurs following CT injury also suggests that microglia are reacting to more than just an interruption in normal neuronal signaling. One possible explanation is that microglia only respond to acute interruptions in normal neuronal activity. P2X-dblKO animals, however, represent a permanent loss of ‘normal’ nerve activity. Future experiments to acutely block activity of the CT nerve and subsequently examine microglial responses will be of interest.

### Minocycline

Although minocycline treatment reduces microglial reactivity in pain models, this treatment did not affect the microglial responses after CT injury. Rather, microglia in the nTS displayed reactive morphologies and increased in number in the RC subnucleus. Even though the number of microglia increased with minocycline, we measured the intensity levels of two microglial markers. While the level of Iba1 was not affected by minocycline treatment, the level of cd11b with minocycline treatment was significantly decreased compared to the ‘No treatment’ group in the RC but only in the R2 rostral level of the nTS. That minocycline did not have a greater effect on microglial responses after CT damage may be an issue of dosage. While the half-life of minocycline is about 15 hours in humans ^38^, its half-life in rodents is reported to be ~3 hours ^39, 40^. Hence, to achieve steady state concentrations more frequent administration of larger doses of minocycline is likely necessary. Nevertheless, the level of cd11b did not significantly decrease in neighboring regions suggesting that the decreased level in the RC subnucleus is a specific effect. The fact that only the levels of cd11b, an intergrin receptor involved in phagocytic activity, were decreased with minocycline treatment is also suggestive that reactive microglia in the nTS are involved in phagocytosis.

### TLR4 experiments

In the central nervous system TLR4 is expressed by microglia and perhaps by astrocytes and endothelial cells but not by neurons ^41^. Although best known as the receptor that detects cell wall components of gram-negative bacteria (i.e., LPS), TLR4 can also respond to “endogenous danger signals” or “alarmins” ^42^. These are signals of cellular stress or disruption including the release of DNA, heat shock proteins and similar components that are normally concealed from immune surveillance. Many such substances are released after peripheral nerve injury, which in turn activate microglia to release a variety of neuroexcitatory and pain-enhancing substances ^43^.

Indeed, recent studies have shown that TLR4 signaling is involved in microglial signaling and related pain behaviors following spinal nerve injuries. Not only does deletion of TLR4 prevent pain from developing after spinal nerve injury ^22^, but inhibiting TLR4 with naloxone can also reverse established pain states ^23, 24^.

***TLR4 mutants (C3H)*:** The role of TLR4 signaling was tested by performing CT injury in TLR4 mutant animals. Following CT damage, microglial cells in the nTS increased in number and were morphologically reactive throughout the CT terminal field in the mutant animals, just as seen in wild type animals. These data suggest that TLR4 signaling is not necessary for microglial reactivity following CT injury. This might suggest that microglia in the nTS may rely on different signaling mechanisms than those described in models of chronic pain.

***Naloxone*:** This treatment has been demonstrated to diminish microglial reactivity in pain models. Interestingly, in addition to binding the classic opioid receptors, naloxone also binds to the Toll-like receptor 4 (TLR4) on microglia ^44^. In the current experiments, naloxone was used to test whether microglial responses in the nTS were dependent on TLR4 signaling. First, to gage whether naloxone was indeed accessing the CNS, the animals’ sweet preference was measured. This is based on a wealth of literature demonstrating that opioid antagonists such as naloxone cause a reduced intake of sweet-tasting substances. The appetite suppressant effect of naloxone is centrally mediated, resulting from neuronal interactions in the reward pathway ^45^. Based on other studies in rats, naloxone caused a decreased intake in sweet foods ^34, 35^. While this hypophagic effect is a result of naloxone binding to neuronal opiate receptors, the behavior is centrally mediated ^46^ and hence indicates that naloxone is accessing the CNS. In the current experiments, animals preferred the sucrose solution during the training period as expected (i.e., preference score > 0.5). After treatment with naloxone, however, the overall sucrose preference did not differ and the animals continue to prefer sucrose. On the whole, the preference for sucrose did not consistently decrease in naloxone treated animals.

While the anorectic effect of systemic naloxone is detectable in a variety of species, including humans ^47^, rodent studies have relied entirely on the use of rats. Herein lies the primary difference between previous studies in rats and the current study in mice. Earlier reports from rats repeatedly show that subcutaneous doses of naloxone from 0.5–10mg/kg/hour caused a significantly reduced intake of sweet tasting food or drink for about 2 hours after treatment ^33– 37^. However, a continuous dosage of 6.96mg/kg/hour did not consistently reduce the sucrose intake in mice in the current study. This may also reflect the difference in the means of administration, i.e., experiments in rats routinely used acute subcutaneous injections of a higher concentration of drug rather than a chronic subcutaneous delivery of a lower dose. Regardless, the current experiments do not provide evidence that naloxone was accessing the CNS at a level sufficient to affect the neuronally mediated sweet preference behavior.

Naloxone treatment also did not affect the microglial responses after CT injury. Rather, microglia in the nTS displayed reactive morphologies and increased in numbers. Naloxone has been shown to affect microglia via an acute subcutaneous administration to rats at 100mg/kg ^23^. This caused full reversal of neuropathic pain behavior as well as decreased expression of the microglial marker, cd11b. Because the half-life of naloxone is about an hour, this acute administration can be roughly compared to the hourly dose received with chronic subcutaneous delivery. However, the highest dose that could be achieved in the current study was 6.96mg/kg/hour, less than ten times that of the acute dose. This is one possibility why microglial responses in the nTS were not affected by naloxone treatment.

However, an alternate interpretation of these data is that naloxone did indeed access the CNS but did not affect the microglial responses. This could be explored by performing a sciatic nerve lesion in mice with the subcutaneous minipumps to deliver naloxone. The chronic naloxone treatment used here can be compared to the effects of acute naloxone, which effectively dampened the microglial response triggered by sciatic nerve injury ^23^. If the subcutaneous minipumps used in the current studies block the microglial responses after sciatic injury, this would suggest that naloxone is accessing the CNS at an effective dose. Further, such a result would argue that signaling mechanisms involved in pain-related microglia reactivity are different than non-pain microglial reactivity, i.e., in the nTS in response to CT injury.

Additionally, naloxone could be administered directly into the brainstem with an intracisternal injection. Subcutaneous administration was chosen in the current study so as not to compromise the blood-brain barrier, a disturbance that can activate microglia. Yet a study in rats experiencing chronic pain used minipumps to deliver naloxone by injection into the intrathecal space (fluid-filled area of spinal cord located between the pia mater and the arachnoid mater). This treatment method successfully reversed pain behavior as well as decreased the expression of microglial markers ^23^. This suggests that naloxone effectively dampened microglial responses that might have arisen from the intrathecal method (namely, making a small hole through the skull and disrupting the blood brain barrier). Using a similar intracisternal injection route of naloxone with CT injury would ensure that naloxone is reaching the brainstem, a question that could not be answered here either in terms of microglial responses or neuronal changes (i.e., taste behavior).

## Summary

Peripheral injury to the CT results in central microglial responses. Even though the CT lacks pain signaling, the general profile of this microglial activation in terms of morphological reactivity and increased density is similar to that seen in nerve injury models of chronic pain ^4^. The current experiments examined several possible mechanisms that could be involved in microglial reactivity after CT injury. Treatment with minocycline, a compound that dampens microglial responses in pain models, was largely ineffective in diminishing microglial responses after CT injury. Also, TLR4 signaling, which is central to microglial reactivity following spinal nerve injury, does not seem to be required for microglial responses following CT injury. Finally, signaling through P2X _2_ and P2X _3_ is also not necessary for microglial reactivity following CT damage, which might also suggest that microglia are responding to more than an interruption of nerve signaling. Taken together, these data suggest that reactive microglia in the nTS rely on different signaling mechanisms than those described in models of chronic pain. Nevertheless, alternative treatment protocols with minocycline and naloxone warrant further investigation.
