# Supplementary material for: Reactive microglia after taste nerve injury: comparison to nerve injury models of chronic pain
Source: F1000Res. 2013 Feb 28;2:65. [Version 1] doi: 10.12688/f1000research.2-65.v1 (PMC3782356; doi:10.12688/f1000research.2-65.v1)
Supplement: Peer review history

# Peer review: Referee response for version 1

**Published:** 2013-04-23

**Authors:** Susan Travers

**Affiliations:** 1 College of Dentistry, Section of Oral Biology, The Ohio State University, Columbus, OH, USA

This paper is an interesting follow-up to the first author’s recent report ( JCN, 2012) that chorda tympani (CT) nerve section causes a robust increase in the number and functional activation of microglia in the rostral nucleus of the solitary tract (rNST), an increase largely confined to the terminal field of that nerve. In the initial paper, the authors established that the mechanism of microglial responses in rNST was distinct from the better-studied response in nociceptive regions of the CNS because of the lack of immune cell migration from bone marrow. The current study provides additional data suggesting that the microglial response in the NST is distinct—it is attenuated only marginally following minocycline treatment and does not rely on the toll-like 4 receptor, again in contrast to what occurs after injury of nociceptive afferents. It is also noteworthy that the microglial response persists in P2X2/P2X3 double knockout mice, suggesting that a lack of gustatory-driven activity does not drive the effect.  

The methods, including the analytical approach, were thoroughly explained or suitable references provided. The experimental design was appropriate and the methods used seemed adequate to test the hypotheses advanced. Because the outcomes were essentially negative, however, it would have been more compelling if an example of a positive control had been presented to show that these authors could replicate the attenuation of the microglial response in the pain system, the system from which these techniques were adapted. On the other hand, the fact that 2 different approaches were used to probe the role of TLR-4 is reassuring. 

The authors’ conclusions are based on the data, which are quite clear, presented in detail, and discussed in an appropriate and scholarly fashion.

I have read this submission. I believe that I have an appropriate level of expertise to confirm that it is of an acceptable scientific standard.

# Peer review: Referee response for version 1

**Published:** 2013-04-08

**Authors:** Lynnette Phillips McCluskey

**Affiliations:** 1 Institute of Molecular Medicine and Genetics, Georgia Research University, Augusta, GA, USA

The authors investigate whether microglial responses to gustatory nerve injury parallel activation in neuropathic pain. This context was helpful as a guide to the experimental progression, as well as a model for their hypotheses.

The experiments were conducted appropriately. Though group sizes were small, the results appeared to be straightforward and could be replicated. A brief rationale for the different behavioural schedules would be useful in that regard.   

The idea that the discrepancy in afferent activity after nerve sectioning (vs. the absence of activity in P2X KO mice) contributes to microglial responses is intriguing and will be interesting to pursue. Distinct receptor subtypes, such as P2X4, may also be involved in microglial responses in the NTS.

I have read this submission. I believe that I have an appropriate level of expertise to confirm that it is of an acceptable scientific standard.

# Peer review: Referee response for version 1

**Published:** 2013-03-08

**Authors:** Odd-Geir Berge

**Affiliations:** 1 Neuroscience, Uppsala University Hospital, Uppsala, Sweden

The results are interesting and worth publishing. I would like to make a few points, however. 

Reporting of methods could have been more complete, there are, for instance, no mentioning of whether any measures were taken to randomize animals to treatment groups or blind the observer to treatments. Neither is there much description of how the animals recovered after the various procedures. 

Applying one of several recently published guidelines aimed at improving experimental design and reporting, e.g. “Kilkenny, C., Browne, W.J., Cuthill, I.C., Emerson, M., Altman, D.G. (2010) Improving bioscience research reporting: the ARRIVE guidelines for reporting animal research *PLoS Biol*. 8(6)”, might have been useful. 

An estimate of the power of the design to detect a reasonable effect of the treatments would have been warranted given the relatively small number of animals in each experimental group. 

Finally, the authors consistently refer to neuropathic model readouts as pain. This is common practice but nevertheless controversial. The models nearly exclusively measure evoked responses to stimuli that may or may not provoke pain and changes in sensory thresholds or perception are not well correlated to clinical pain. A more stringent vocabulary would make interaction between different professions easier.

I have read this submission. I believe that I have an appropriate level of expertise to confirm that it is of an acceptable scientific standard.
